# Narratives of Natural Recovery: Youth Experience of Social Inclusion through Green Care

**DOI:** 10.3390/ijerph110606052

**Published:** 2014-06-06

**Authors:** Ragnfrid Eline Kogstad, Rita Agdal, Mark Steven Hopfenbeck

**Affiliations:** 1 Department of Public Health, Hedmark University College, Box 400, 2418 Elverum, Norway; E-Mail: rita.agdal@gmail.com; 2 Department of Health Care and Nursing, Gjövik University College, Box 191, 2802 Gjövik, Norway; E-Mail: Mark.Hopfenbeck@hig.no

**Keywords:** Green Care, youth social inclusion, recovery

## Abstract

The aim of this study has been to investigate the effects of Green Care services for youth in vulnerable situations risking social exclusion. Green Care enterprises represent alternative arenas in which people can work with animals, agriculture and other tasks related to nature. We interviewed nine persons, aged 17–27, working in three different places, two or more times over a two-year period. We looked at essential beneficial factors in order to better understand how the “green” element could add to more traditional recovery factors. We found that the youth described core success factors corresponding to well-known recovery factors such as recognition, supportive relationships, motivation, meaning, positive coping, self-esteem, confidence and hope. The effective factors can be described as: (a) The leader’s ability to create a good group atmosphere, (b) the varied tasks which allow step-wise increases in self-efficacy, and (c) experiences with animals and in nature that provide comfort for youth who lack trust in people and need safe situations to recover a positive sense of self. We followed a process in which several persons gradually regained self-respect and the motivation for further education or a job outside the Green Care enterprise. The study illustrates that Green Care can be an important supplement in helping people back to a satisfying life and meaningful roles in society.

## 1. Introduction

In this article, we present findings from a study conducted at three “Green Care” enterprises, in which young people, aged 17 to 27 years, who were not at school or in work, were occupied with tasks related to nature, animals and agriculture.

Marginalization and social exclusion among young people are related to several factors. Labour market forces and national economic policy play a major role [1], as well as features of the school- and welfare systems, and whether there exist alternative, flexible and person-centred pathways and models of care for young people, who for different reasons do not finalize their education along the standardized route.

Dropping out of school increases the risk of social exclusion. Between 8%–26% of young people between the ages of 20 to 25 years in the Nordic countries have not fulfilled their upper secondary education. As a result of different compensating welfare strategies, however, only 3%–8.5% are not in education, employment or training; the so-called NEETs [2]. The OECD average percentage of students who enter an upper secondary programme for the first time, and who graduate from it, exceeds 80% when calculated as the relationship between the graduates and the new entries in the same level of education. Norway and Denmark have a slightly better result than the OECD average, while the Swedish result is lower than the OECD average [3]. In the NEET group we find people who have dropped out for a period in order to travel and focus on self-development, or whom intentionally wish to distance themselves from society. However, the largest proportion of the NEET group is unemployed. In other words, up to 30% drop out before or during high school, less than 10% seem to end up without any employment, education or training, and between 5%–10% of young people in the Nordic countries seem to risk permanent marginalization from employment and education [1]. The cost for one person dropping out of school is estimated at 9,000 EUR [4]. In addition, there are social expenses related to lower tax income and the possible consequences of anti-social behaviour. In economic terms one can argue that spending up to 9,000 EUR on one person will be profitable as far as this person is enabled to finish the upper secondary programme [2]. The challenge is both to find solutions for this relatively small group and protect more persons in the 30% group against the risk of social exclusion.

Efforts to prevent drop out and social exclusion include both attempts to motivate the students while they are in the school system and activities in alternative arenas aiming at supporting self- confidence and competence in safe environments [5]. In this article, we focus on such alternative arenas and restrict the investigation to the relatively small portion of enterprises which offer Green Care-related employment schemes, in order to examine whether the participants experience that the “green” or natural element adds something extra to the recovery process.

Green Care is a well-established international concept, and is defined as the “Utilization of agricultural farms—the animals, the plants, the garden, the forest, and the landscape—as a base for promoting human mental and physical health, as well as quality of life for a variety of client groups” [6]. Green Care implies an active process intended to promote health and well-being in natural environments, including the therapeutic use of green environments and nature in general, as well as farms, gardens and animals [7]. In general, studies have shown positive changes related to physical health, stress reduction, social abilities and general condition, in addition to self-confidence, problem-solving ability, experienced meaning and responsibility [6,8,9,10,11].

### 1.1. Recovery Theory as an Approach to Understanding Mechanisms Promoting Social Inclusion among Young People at the Risk of Marginalization

The evaluations of strategies to reduce drop out, increase employment and strengthen social inclusion show varied results, partly because of the difficulties in identifying methods or strategies which will work for all. A more realistic aim may be to find out why something works for a particular person [12].

Still, different qualitative studies have pointed to certain core success factors: close individual follow-up, individual adaption and facilitation, interdisciplinary cooperation where the social network is included, a holistic approach and unbureaucratic structures which invite contact and quick response [5]. Fröyland [13] has added the anchoring of strategies at different administrative levels, dedicated entrepreneurs, varied tasks which open up possibilities for successful coping on different levels and arenas, and neutral arenas for meetings between the help service system and the youth. Additional factors identified in several studies [14,15,16] were time, stability and continuity.

Essential helpful factors discovered in the aforementioned studies among young people at risk of marginalization overlap with factors promoting health and social inclusion in recovery literature, with a focus on existential, functional and social recovery, as opposed to medical recovery, in which the reduction of symptoms is a central criterion. The recovery concept in this context first points to the fact that people can recover from psychosocial disabilities, and second that the means are universally human such as respecting and appreciating the person’s individual solutions, supporting hope and motivation, building good relationships and networks, promoting citizenship and strengthening identity, dignity, meaning and efficacy [17,18,19,20,21,22].

A trait found among young people who drop out of school is their feeling of not being appreciated for who they are [16]. The school system is marked by competition that may never have been intended to make groups of persons feel less worthy as human beings, but this feeling is nevertheless being produced among students. The feeling is being handled in different ways. A study by Skaalvik [23], illustrated how low marks led to the students doing less homework so that they could accuse their lack of effort when they did not succeed in school. This seemed easier to live with than the feeling of being an incompetent person, unable to reach the level of those who achieved the most.

It is a challenge to create arenas in which young people can feel competent and achieve recognition. According to Honneth [24], recognition means to see the person and be with the person over time, as a lack of recognition may cause infringement. But Honneth emphasizes that recognition is not only a micro-phenomenon, it also includes justice and redistribution at the macro level, which is important to bear in mind when we examine how young persons can regain self-esteem and a motivation to change.

Research on relationships between nature and humans has pointed to beneficial effects from interaction with natural environments such as stress reduction, positive social contact and physical activity [7,8,10,25,26]. We still need to understand more about the way nature-based services may produce health benefits and recovery. Simple cause-effect studies will not provide all the answers, as it is necessary to understand how different factors work together to produce the desired results [8,10,11].

### 1.2. Aim

The aim of this study is to investigate the presence of recovery factors in Green Care enterprises, and how these factors can promote social inclusion. We focus on Green Care enterprises that offer employment schemes for youth to improve their opportunities for entering the work force or aid them in continuing their education. We have a special focus on what the “green” element may add to other, more well-known recovery aspects, and in which way this element may eventually help to facilitate recovery processes.

## 2. Methods

The data material stems from observations and qualitative interviews at three enterprises where adolescents participate in employment schemes financed by the labor and welfare sector. The enterprises were located in different parts of Norway. Since the role of recovery factors in Green Care has not been extensively studied previously it was decided to carry out in-depth field work at all three locations in order to better understand the processes involved. Individual interviews with three youths from each enterprise were also carried out. In addition, we had informal talks with all the leaders and conversations with other participants who had consented to us visiting them, but did not wish to be interviewed. All the participants in the study had initially dropped out of school and had traumatic experiences related to feelings of failure and exclusion in school. Apart from this, there were different degrees of severity in their situation related to problems with substance abuse, self-destructive behavior and suicide attempts in the near past, as well as lack of stable adults in their childhood and traumatic life events. Common traits were experiences related to isolation, inadequacy and a feeling that the future might be lost.

The enterprises were selected from lists provided by the regional agricultural authorities. The three enterprises were chosen to provide variety, as they were located in different municipalities and had different kinds of agricultural production which we assumed would provide different arenas and activities for the participants. These employment schemes do not follow a standardized program and do not lead to any formal qualifications, but the participants work at the enterprise approximately six hours a day, including lunch break, five days a week. They had chosen to take part in the employment scheme after discussing options like ordinary work or education with an advisor at the labor and welfare office. The participants in these schemes are entitled to additional support from a consultant from the labor and welfare office. All the enterprises that were asked to participate agreed to be part of the study. All the leaders had several years’ experience with working with at-risk adolescents, but no formal competence as health or social workers. The leaders can be described as entrepreneurs as Green Care activities are encouraged by the agricultural department as a diversification of farming.

It was the youths’ own decisions to take part in Green Care arrangements and they all seemed to have an inner motivation for the work. At the same time other opportunities may have been absent in that region at that moment and participation may have been perceived as one of very few existing options.

The leaders passed on envelopes with invitations to participate in the study to all adolescent who were engaged in employment schemes at their enterprises. The adolescents could choose to participate in this project by returning a signed letter of consent.

The interviews were semi-structured, covering seven topics listed in an interview guide; expectations, flexibility in the daily programme, relationship to the leader, relationship to group members, contact with animals and nature, cooperation between different help services and thoughts related to alternative opportunities if the enterprise had not been there for them. The topics were chosen on the basis of earlier research where essential factors in Green Care services are outlined [27].

Each one of the three researchers was responsible for one enterprise, but we also visited each other’s places and discussed similarities and differences. During the interviews, we alternately used a tape recorder and made notes. Two of the informants read through the interviews with them based on written notes, accepted the citations and found that the content was in line with what they wanted to impart.

The data material was analyzed according to a qualitative content analysis [28], and was read through several times by the researchers, to identify and code experiences which stood out as positive or negative experiences as they were described in the interviews. The texts segments that were identified as descriptions of positive or negative experiences were categorized. In this process, the researchers proposed categories independently of each other and negotiated the final categories, before the categories that emerged were related to recovery theory.

### 2.1. Informants

The participants who consented to the interviews were interviewed two-four times, with the time span for contact with each of them varying from two months to almost two years. The intention was to follow each participant for one year, to follow transformational processes. However, we found that not all participants took part in the employment scheme for one year, as this was adjusted according to personal needs and negotiations with the labor and welfare sector. The difference in time is related to the variation of duration of the employment schemes, as some continued with other activities after a while and some were allowed more time to participate in the scheme. A standard period seems to be one year with opportunities for an additional year, but as explained above, there were also variation related to personal needs and preferences. Table 1 shows the length of the interview period for the different participants.

**Table 1:** Time schedule for the interviews.

Name, Age	1st Interview	2nd Interview	3rd Interview	4th Interview
Lynn 25	January 2012	June 2012	January 2013	
Therese 25	January 2012	June 2012	June 2013	
Ruby 25	June 2011	November 2011	January 2012	May 2013
Vida 27	November 2012	January 2013		
Nicolas 25	April 2012	October 2012	December 2012	
Mary 20	April 2012	November 2012		
Olga 17	January 2011	January 2012	May 2012	
Stuart 19	September 2011	March 2012	November 2012	March 2013
Ann 20	November 2011	January 2012	May 2012	

We met the participants at different stages. Some had just entered the enterprise, while others were close to leaving and proceeding to other activities. The material does not allow exact comparisons between groups who started at the same time, under the same conditions, at the same age and with the same problem description. Such material could hardly be found in this group because all the individuals had different processes. When it comes to Green Care services, such comparisons are even more difficult because the services are so varied and the success criteria seem to be composed of several elements such as the leader’s personality, environment, activities, group characteristics and support system outside the enterprise. Even so, it is still possible to study recovery processes and identify possible core success factors, and compare these to findings in other studies.

### 2.2. The Green Care Enterprises and the Programs

The first enterprise was originally planned as a Green Care farm, but the welfare office did not provide the farmer with sufficient economic support. Instead, he was offered a job as a group leader in a sheltered workplace—more like a factory—close to his farm. Here, he worked as a group leader, gave the youth adequate work tasks and also regularly brought them to his farm, where they could be together with his dogs and horses. Additionally, he took them on other trips, both by mini-bus and horse-pulled wagon, and in total there were six places in the youth group.

The second enterprise was a large farm with horses, dogs, cats, pigs and rabbits. The farmer was the leader and responsible for all planning, and he had an assistant who took care of the youth and followed them up. The farm had many visitors and received school classes and institutions. The youth were made responsible for hosting the visitors and organizing activities. To some degree, the youth could choose their activities, but there were some “musts”, such as feeding and caring for the animals. At this enterprise there were six places in the youth group.

The third enterprise was a small farm with several small buildings, and the place was run by a husband and wife who had several animals. They had a riding school, and offered activities for kindergartens, elderly people and older school children. Three persons had permanent sheltered jobs in the farm financed by the labour and welfare sector. Moreover, the farm received a limited number of young people for shorter or longer stays on an individual basis.

The content of the programs is most of all characterized by variation and flexibility, and also adapted to the different seasons. Breakfast and lunch together are standard. During the meals there are open, personal conversations about health conditions, recent experiences and personal preferences regarding activities and how much work the individual participant felt ready to perform. Partly the program of the day is set by these talks taking place during the meals, and partly by necessary tasks that must be attended to, like feeding animals, cleaning the stable, weeding the vegetable garden, splitting firewood, *etc.* The youths are accompanied to dentist, doctor or the labour and welfare office when this is needed. And gradually, if they are ready, one or two days a week may be spent in school or in a workplace. All of the enterprises received funding from the labour and welfare sector and the leaders/farmers cooperated, with varying degrees of success, with a number of youth-related services outside the farm.

In addition to the interviews, we had informal talks with the leaders and other participants, and we found that not all identified with the farm or felt that they fit into the concept. There were people who preferred to leave after a while, whereas others were not able to solve their substance abuse problem. These were not automatically rejected, but if they were not willing or able to work with the problem after some time (several months), it was felt that the farm was not the right place for them because of the risk connected with handling big machines and the effect their substance abuse had on the rest of the group. The information we got may indicate, which is probably true, that farm work and farm life does not fit everyone, but this is also dependent on how social relationships are nurtured and developed, as well as on the activities. The activities on Green Care farms may not necessarily be restricted to traditional farm work, but may also include among others handcrafts, mechanical tasks, artistic work and meditation.

### 2.3. Ethical Issues

The project was accepted and registered at the Norwegian Social Science Database (NSD), which has an authority delegated from the Data Inspectorate of Norway to accept investigations in which sensitive, personal information is involved. Participation was based on free and informed consent, and the participants were not contacted directly by the researchers. Enquiries about interviews were sent via leaders of the enterprises, and it was up to the youth to sign the enquiry form and send it back to the researcher. The right not to participate, and to also withdraw at any point during the study, was underscored. The participants were ensured that their right to privacy and integrity would be maintained and that no information would be given that would reveal the participants’ identity when the results were published. The interview guide focuses on the youths’ own opinions about the activities, which factors have been the most important to them, changes in their life situation and what they think about the future.

## 3. Results

In Table 2, we use direct extracts from the interviews to present a brief overview to help illustrate essential ingredients in the change processes and achievements during the process. The table is based on an analysis where we categorized elements from the interviews. We did choose information related to active elements in the recovery process (left side) and remarks related to what they obtained during the process (right side).

**Table 2:** Important elements in the process and achievements.

Name	Essential Elements in the Process	What Has Been Achieved
Lynn	That the leader does not condemn you, but listens, advises you in a straightforward way, and finds tasks you can master. And that you can look forward with positive expectations to each day.	I have learned self-discipline and developed self- esteem. Can see the brighter sides of life. Have gotten a practice place and started school again. The aim is to qualify for a job where I can work with people.
Therese	The leader creates safe environments. He is honest, and has a role like that of a father role. He has taught me skills I missed in my childhood. Looking forward with positive expectations to each day.	I have learned to finish tasks—because I experience that I can master more than I believed before. Have learned to stand up for myself. Have returned to school and succeeded. Will study and become a social worker.
Ruby	Was very well welcomed. The leader never abandons or judges us, even if we fail. He trusts and respects us. He has clear values. Important: Time for talks, the group, the leader, the animals.	I have become more social. Will soon finish high school. Received top marks and will study and have a professional career where I can help young people and make sure they are not forgotten when they struggle.
Vida	Has been pushed a little bit and received support and acceptance. Can also be on my own. The leaders see the person and have much insight. Do not judge. Important: Times for talk, the group, horse riding.	I have learned about myself and how to handle both people and animals. Animals can give you confidence and make you feel safe. Have learned to plan and set realistic aims. It is not stigmatizing to work on a farm.
Nicolas	Talking to the leader, who has rich personal experience, is better than talking with professionals. He is very accepting. Important: The good community and support from group members, farm work.	Feeling of competence by helping others. I have learned horse pedagogy. Enjoy being outdoors. Would like a total of two years at the farm. Have been helped through mental health problems caused by dramatic life events.
Mary	I love animals and thrive at the farm. Like to be on me own and together with the animals. Doing my own things.	The farm has rescued me. Thriving together with the animals.
Olga	Says that leader decides too much, but they also thrive together. The leader has praised her for her work. Would like to work fulltime with horses. Can master horses and understands them.	I enjoy being together with the other participants. Am supervising and helping visitors. Have learned to admit failure and endure situations that are experienced as threatening. Will soon be ready for a job.
Stuart	Admires the leader and appreciates that they celebrate birthdays and have meals together. Appreciates all opportunities to talk as a way to develop, and is proud of his position in the stable. Feels responsible.	I have developed regarding personal hygiene, social and practical skills. Have a personal relationship with the animals, and cares about both animals and humans. Would like to work at a farm. Have gotten a permanent job there.
Ann	Appreciates the leaders’ kindness and confidence in her. Thinks that this place is perfect. Important: Varied tasks, being competent, being alone, feeling peace, the group, the horse that accepts you.	Experience it is easier to think and find back to myself when I can be alone - like a cloud disappearing. Can take care of myself and others. Feel safer and stronger. Feeling of confidence when working with horses. Will go on with school and is soon ready for living in my own flat.

Key words for the participants’ achievements during the stay are motivation, a feeling of peace, self-discipline, realism, self-esteem, self-insight, self-care, optimism, ambition, ability, independence, confidence, strength, recovery from mental health problems and a feeling of being more social, as well as return to school or a job.

To some degree, the results mirror both the length of their stay at the enterprise, but also the conditions they brought with them when they started. In Table 3 we compare the length of stay when they reported their results to us the last time to what they had achieved.

**Table 3:** Time at the farm and personal achievements (briefly described).

Name and Age	First Time at Farm	Last Interview	Achievements
Lynn	June 2011	January 2013 2 years later	Self-discipline, optimism, motivation, self-esteem, practice place, return to school
Therese	November 2011	May 2013 1.5 years later	Self-discipline, competence, independence, self-confidence, motivation, ambition
Ruby	February 2011	May 2013 2.5 years later	Has become more social and confident, motivation, ambition, self-esteem, independence, strength
Vida	August 2011	January 2013 0.5 year later	Has gotten self-insight, realism, confidence, ambition, self-care
Nicolas	May 2011	November 2012 1.5 years later	Self-efficacy, recovery from mental health problems
Mary	May 2011	November 2012 1.5 years later	Feeling of peace, recovery
Olga	November 2011	May 2012 1.5 years later	Competence, motivation, ambition, self-insight, become more social
Stuart	November 2010	April 2013 2.5 years later	Competence, care and self-care
Ann	November 2011	November 2012 1 year later	Self-insight, self-esteem, self-care, strength, competence, motivation, feeling of peace

Among the three who were interviewed two years or more after they started, one had gotten a permanent (sheltered) job at the farm, and two returned to school and are on their way to a professional career. In the group who had been there for 1.5 years, one had returned to school and succeeded very well, while three had made much progress related to competence, hope and trust. Two persons had been in the place for six months to one year, and they both stressed the importance of experiencing peace, being on their own and accomplishing different tasks.

The material indicates the positive development that is possible over a period of one to two years, although the process is step-wise and we need to be aware of the “on the way” results as well as endpoints. Self-confidence, a feeling of calmness, acceptance and hope [17,18,19,22] are all conditions for work and educational success and society’s demands in general, and should be seen as important results in themselves. Furthermore, we know that not all may be able to cope with demands in today’s work life or the school system, so factors such as self-confidence, acceptance and hope will be even more important as endpoints.

Even though all our informants report positive changes, they also were aware of the possibility for improvement. There are differences between the enterprises, and some may have advantages that are missed at others. For example, the balance between activities with animals and in nature on the one hand, and the close personal following up on the other, will differ. Some of the participants were followed up more closely than others by advisors outside the scheme, although there was not much contact between these advisors and the leaders at the farms. For those who were in contact with many advisors from the health sector and the labor and welfare sector, we observed that the advisors lack of knowledge of Green Care created some tensions and misunderstandings. In one instance, what the scheme referred to as “work” was often seen as a mere “activity” by the advisors, which bore different connotations and implied different obligations for the participants. The participants also referred to their participation in the scheme as “going to work”, and protested when others referred to it as “an activity”. The understanding of the scheme as “work” implied pride and an obligation to get there every day, as well as to take on tasks that were important and needed to be done.

When Green Care did not work so well for the young people, it seemed to be primarily due to a lack of coordination and cooperation between the enterprise leader and other help services, including the transition from the enterprise to other forms of work, education or support.

The relative importance of different factors is elaborated on in the next section, in which we discuss the main categories emerging from the informants’ stories about what was most helpful in their recovery process.

### The Important Elements seen from the Participants’ Perspectives

Table 2 illustrated how some themes were recurring in the interviews. In our initial analysis we identified the following general elements the participants considered to be important; safe environments, absence of judgments, acceptance and recognition, meaningful and pleasurable tasks that allowed the participants to experience self-efficacy and competency, looking forward to the next day with positive expectations, honesty, clear values, leaders that to some degree take a father and mother role, time for talks, trust, respect and kindness, a good atmosphere in the group, to be pushed a little bit but also have the freedom to withdraw, varied tasks, animals that accept you, the farm work, experiencing silence and feeling peace.

The elements may be summed up in three main categories: The leader and the group atmosphere, the building of self-efficacy through individually-adapted meaningful tasks, and the animals and nature.

#### (a) The Leader and the Group Atmosphere

The leader’s ability to create a safe environment, marked by recognition, kindness and honesty, is emphasized by almost everyone. The group atmosphere is also essential and means that social networks are developed, but if it was not for the leader, the group would not function:

By itself, the group is the most important element, but it’s the leader’s merit that makes it works. He created the good atmosphere that enabled the group to have such a supportive role.

*—Therese*

It is underscored that the leader must be respected, but the respect must not be based on fear. If he or she combines the ability to give feedback with sufficient safety and support, then the youth experience growth. As expressed by another:

I needed this one person to go on in life.

*—Ruby*

Most often, it is the personal qualities by the leaders and their personal experiences that are emphasized, and that they have experienced hardships themselves and have in-depth knowledge about- and an engagement in the group they are working with.

The qualities of the leader that are appreciated by the youth are also his/her ability to secure time and stability. In addition the youth underline the importance of being together in neutral arenas:

*The leader is always available, we can call him day and night. His generosity creates a feeling of safety and we don’t need to violate his trust*.

*—Ruby*

I would like to stay in this place one more year. I am not yet ready for other things.

*—Lynn*

Travelling by a horse-pulled wagon means opportunities for long talks.

*—Ruby*

It has been important to escape the stigmatization following being a care receiver. In this place I am employed, that is different.

*—Olga*

As we see, doing a regular job counteracts stigmatization and creates dignity. But a strong job-focus may also mean that the arena which facilitates self-development is threatened, as we have illustrated earlier. The clear message is that having a job supports the feeling of dignity, but only as far as the job is meaningful and helps the person develop positive coping and a sense of competence.

#### (b) Building Self-efficacy through Individually-adapted Meaningful Tasks

In farms and farm-like places there will be varied tasks, which then make it possible to find tasks for all levels of proficiency. A good leader seems to stimulate self-efficacy in a step-wise process:

I lack self-confidence. But this neither means that he takes over, nor that he gives me tasks I can’t manage. He is encouraging and supervises me in the right direction.

*—Lynn*

It is important to come up with different things, and that the tasks are varied.

*—Ruby*

The most critical element in my process is that my confidence in my own abilities has been built up. But now I have experienced that I can manage more than I believed. I have gradually learned to trust my abilities.

*—Therese*

In addition the informants underlined that it feels good to be physically worn out and to also experience that the job is valuable.

The process from losing self-confidence to dropping out of school was very well described by one of the informants:

I was shy, and did not dare to raise my hand and talk in front of the others. Then you are easily stigmatized as bad and the teachers do not bother to follow you up. They are not good enough in helping you back on track. The school should not be such a competition system; it is something about these marks. You get stigmatized as bad, then you don’t bother and the teacher does not take the trouble.

*—Ruby*

She is now finishing upper secondary programme and is doing pretty well.

#### (c) Animals and Nature

As expressed by many: Animals are very important when you don’t trust people. As stated by Vida:

The animals do not condemn you.

*—Olga*

The young people elaborate on this by talking about how they could give the animals something of value and see that they thrive. Some of the participants have grown up with animals and outline how they looked to the animals when they were sad, and were encouraged by them.

The natural arena is also highlighted. Some participants talk about the silence they find in nature and about opportunities to be on their own:

It’s like a cloud disappearing.

*—Olga*

Working in the stable can also give a certain status in the group, as being the boss in the stable means to be acknowledged at the farm.

Nature is an arena for social contact and good talks: Sitting around a fire in the forest or travelling by a horse-drawn wagon means unique opportunities for talks about personal and important issues. One of the leaders pointed to these situations as opportunities for the youth to clear their heads.

## 4. Discussion

As mentioned in the introduction, different qualitative studies have identified some core elements that have been proven to be essential when young people are helped back to school and employment, and develop self-confidence, hope and competence. The factors involve adapting care to individual needs, dedicated entrepreneurs, varied tasks, neutral arenas, time and stability [5,13,14,15,16].

All of these aforementioned factors are recognized in our material. Summarized, they can be described as the intention to build motivation and self-efficacy by supporting self-esteem, self-insight, self-care, confidence, hope and optimism. Recognition emerges as a fundamental value that supports the individual’s feeling of dignity [21], and which becomes distinct when contrasted with competition and disciplining as a means of socialization. Well-known core elements in recovery processes such as good relationships, meaning, positive coping and citizenship [20] also emerge as outstanding features when the youth describe their way back to a more satisfying life in which they can imagine having a meaningful role in society.

Enterprises established to give young people an opportunity to recover and find their way in life are not standardized projects. At present, such undertakings are more like pilot projects that will vary in quality and orientation. In the longer term, there is a need to refine more of the effective elements and see how they may be systematically combined and uniquely implemented within a given context determined in part by the individual enterprise leader [27].

Nevertheless, our findings combined with other studies of alternative employment schemes for young people [2,5,14,29], contributes to a preliminary sketch of “the good enterprise”: The value system is based on recognition rather than disciplining and competition, the leader is a warm, dedicated person with comprehensive life experiences and a personal engagement in each one of the participants, there is time for talks and the arena is safe and neutral, the working tasks are varied and meaningful, the group atmosphere is positive and encouraging, there are network meetings with family- and help service officials and sometimes representatives from the school system and the labour market, and that the youth are also followed up after they have left the scheme. Our findings also indicate that it is important that advisors in the labor and welfare service as well as the health service have knowledge about Green Care in general and the given scheme, so that the participants efforts are acknowledged.

Regarding activities and work tasks, farms can potentially open up for a range of different activities. But it is also possible to build networks and establish coordination between farms and other enterprises in order to offer the youth a broad spectrum of opportunities, thereby enabling them to develop confidence and self-esteem in areas where they have individual strengths and interests.

One of the main questions in this study is about the “green” element: What do nature and animals add to enterprises aiming at restoring self-esteem and self-efficacy in young people? As we have seen, there are three elements that were of particular importance for several participants: To relate to beings who do not judge them, to learn that animals are dependent on them, and appreciate their care and feeding, and to experience silence and acceptance in natural environments. Kaplan [8] has worked with Attention Restoration Theory for years, providing an analysis of the kinds of experiences that lead to a recovery from the attentional fatigue resulting from information-processing demands. He points to the natural environment as being particularly rich in the characteristics necessary for restorative experiences. Directed attention is seen as a key psychological resource in order to cope with challenges, and it is here according to Kaplan that natural environments may play a powerful role. Irvine and Warber [9] argue that as definitions of health and healing broaden and we understand more of the interconnection of mind, body and spirit, it is time to reconnect people with the natural world and draw benefits from the healing powers of natural environments. For example, their literature review shows that a field with trees is a more preferred setting than one without trees, and that such preferences show a remarkable consistency across both demographics and landscapes. They also found that green outdoor settings were perceived as being significantly better than indoor activities or activities in man-made settings and that the relaxation response after the interaction with nature was found to be similar to that elicited by various forms of meditation. These findings are supported in Myrvang’s study [30], in which physical activities in natural environments had better effects for the participants’ mental health than just physical activity. Bele [31] attempts to explain this finding by the presence that nature invites, a presence in which human beings can connect to their own existence and feel authenticity [32], and compares this presence to what is experienced during mindfulness training.

An interesting question, not focused on in the interviews, is whether the young people have had contact with nature and animals in the past, and thereby were more likely to connect with nature and animals in the present [33]. From participant observation and informal talks we learned that all the youth in our material had positive experiences related to animals and/or nature in the past, which may explain the high levels of satisfaction they reported. Our material indicates a probable link between experiences in the past and beneficial effects of Green Care, but the connection should be further investigated.

## 5. Conclusions

Social exclusion among young people is a serious political concern, as are the fragmented services with their lack of predictability and the unstable financial situation in the field [34]. In this qualitative investigation, we have studied three enterprises where young people who are not yet ready for further school or a traditional job have received the opportunity to develop confidence and a belief in their own abilities as a step on their way back to education and employment, or simply a more satisfying life. The study confirmed central elements found in other studies about alternative qualifying undertakings, and illustrates some universal recovery principles. A core element seems to be that the participants are recognized as individuals and respected. The enterprises are not marked by competition, nor are the participants seen with a disciplining or pathologizing eye. Competition seems good for the winners, but not the losers, as illustrated by the young woman (see results) who described the feeling of stigmatization and how this led her to give up.

The sad part of the story this young woman was a part of is that the enterprise she entered no longer receives funding for special youth groups. Political guidelines seem to be interpreted by the local labour and welfare sector as if discipline and production are the most important end goals, which also mirrors the fragmentation and lack of predictability in this field.

We do not claim to have discovered what the ideal enterprise should look like, but our findings support results in several other investigations focusing on elements important in order to recover and enjoy both personal growth and social inclusion. In addition, we have seen that nature and animals can add important qualities in the recovery process.

There are indications that a connectedness with nature may be a more central matter in our time, following the biophilia hypothesis [35,36], and detachment from the natural environment is an increasing problem. However, the ability to connect with nature may differ depending on childhood experiences [33]. If this is so, it is important to allow children the ability to build up the connection to nature so that they later in life may use nature as a resource in recovery processes.

The study emphasizes that Green Care- and nature-based services can be a very important supplement to traditional health- and welfare services, and can help people back to a satisfying life and meaningful roles in society.
